# Ablation of C-type natriuretic peptide/cGMP signaling in fibroblasts exacerbates adverse cardiac remodeling in mice

**DOI:** 10.1172/jci.insight.160416

**Published:** 2023-07-10

**Authors:** Franziska Werner, Estefania Prentki Santos, Konstanze Michel, Hanna Schrader, Katharina Völker, Tamara Potapenko, Lisa Krebes, Marco Abeßer, Dorothe Möllmann, Martin Schlattjan, Hannes Schmidt, Boris V. Skryabin, Katarina Špiranec Spes, Kai Schuh, Christopher P. Denton, Hideo A. Baba, Michaela Kuhn

**Affiliations:** 1 Institute of Physiology, University of Würzburg, Würzburg, Germany.; 2 Institute of Pathology, University Hospital Essen, University Duisburg-Essen, Duisburg, Germany.; 3 Interfaculty Institute of Biochemistry, University of Tübingen, Tübingen, Germany.; 4 Core Facility Transgenic Animal and Genetic Engineering Models, University of Münster, Münster, Germany.; 5 UCL Centre for Rheumatology and Connective Tissue Diseases, Royal Free Hospital, London, United Kingdom.

**Keywords:** Cardiology, Cyclic nucleotides, Fibrosis, Peptides

## Abstract

Excessive activation of cardiac fibroblasts (CFs) in response to injury provokes cardiac fibrosis, stiffness, and failure. The local mediators counterregulating this response remain unclear. Exogenous C-type natriuretic peptide (CNP) exerts antifibrotic effects in preclinical models. To unravel the role of the endogenous hormone, we generated mice with fibroblast-restricted deletion (KO) of guanylyl cyclase-B (GC-B), the cGMP-synthesizing CNP receptor. CNP activated GC-B/cGMP signaling in human and murine CFs, preventing proliferative and promigratory effects of angiotensin II (Ang II) and TGF-β. Fibroblast-specific GC-B–KO mice showed enhanced fibrosis in response to Ang II infusions. Moreover, after 2 weeks of mild pressure overload induced by transverse aortic constriction (TAC), such KO mice had augmented cardiac fibrosis and hypertrophy, together with systolic and diastolic contractile dysfunction. This was associated with increased expression of the profibrotic genes encoding collagen I, III, and periostin. Notably, such responses to Ang II and TAC were greater in female as compared with male KO mice. Enhanced Ang II–induced CNP expression in female hearts and augmented GC-B expression and activity in female CFs may contribute to this sex disparity. The results show that paracrine CNP signaling in CFs has antifibrotic and antihypertrophic effects. The CNP/GC-B/cGMP pathway might be a target for therapies combating pathological cardiac remodeling.

## Graphical abstract

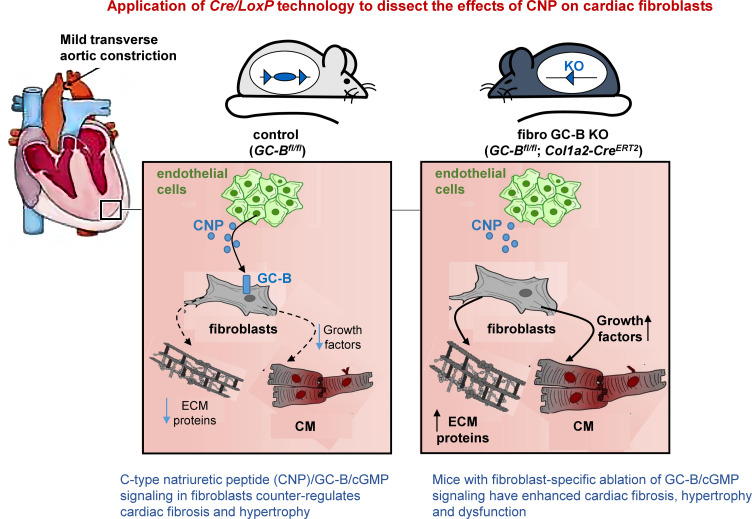

## Introduction

Enhanced cardiac fibrosis in response to acute or chronic injury hinders the efficient therapy of heart diseases. The involved factors, such as TGF-β and components of the renin–angiotensin II (Ang II)–aldosterone system, stimulate the proliferation of cardiac fibroblasts (CFs) and their differentiation to secretory myofibroblasts. Accumulation of noncontractile extracellular matrix (ECM) proteins, especially collagens, increases passive stiffness and the hemodynamic burden on the remaining cardiac muscle (1). Cardiac contractile function declines, eventually leading to heart failure. The knowledge about endogenous factors normally counterregulating these profibrotic pathways is limited and mostly derived from in vitro research. Improvement of this knowledge could support the development of novel therapies.

C-type natriuretic peptide (CNP) is a paracrine-acting hormone with essential functions in chondrocyte-mediated long bone growth (2). In the cardiovascular system, endothelial CNP complements the beneficial actions of the endocrine heart hormones atrial natriuretic peptide (ANP) and B-type natriuretic peptide (BNP) by locally moderating vessel tone and stabilizing endothelial barrier functions (2). In the heart, CNP is expressed in coronary endothelial cells and, albeit less, in cardiomyocytes and fibroblasts (2, 3). Levels are upregulated in clinical and experimental heart disease (3). Infusions of synthetic, exogenous CNP or of stabilized peptide mimetics had protective antihypertrophic and antifibrotic effects in preclinical models of pathological cardiac remodeling, for instance in experimentally induced myocardial infarction (4). Moreover, mice with cardiomyocyte- or fibroblast-specific CNP gene deletion developed increased cardiac hypertrophy and fibrosis in response to left ventricular (LV) pressure overload (3). Studies with cultured CFs indicated that such antifibrotic actions are mediated at least in part by direct inhibitory effects of CNP on fibroblast activation (5). In accordance, exogenous CNP administration exerted antifibrotic actions in experimental lung, liver, and kidney diseases (6–8).

While such pharmacological and genetic studies concur with the notion that CNP inhibits cardiac fibrosis, there are conflicting reports about the receptor mediating this effect. Human and mouse genetic studies unambiguously demonstrated that the critical roles of CNP in bone are mediated by the specific guanylyl cyclase-B receptor (GC-B, gene name *Npr2*), forming cGMP as intracellular second messenger (9). Besides activating GC-B, CNP binds with high affinity to a second natriuretic peptide receptor type C (NPR-C, gene name *Npr3*), which lacks a guanylyl cyclase domain (2). Mice with global NPR-C deletion have larger bones and mild hypotension, indicating that NPR-C is a clearance receptor that captures and thereby terminates the actions of NPs (10). Other studies showed that binding of CNP to NPR-C can trigger G protein–linked processes and thereby modulate cell functions (2, 11). Whether and how the same receptor either clears NPs or mediates their cellular effects is enigmatic.

Studies in vitro showed that CFs express both CNP receptors, GC-B and NPR-C (3). Intriguingly, the profibrotic cardiac phenotype of mice with cardiomyocyte- or fibroblast-specific CNP inactivation was reproduced in mice with global NPR-C deletion, but not in mice with systemic GC-B deletion (3). Therefrom, the authors concluded that the antifibrotic actions of CNP are entirely mediated by NPR-C. However, these global GC-B–KO mice have a complex systemic phenotype, with severe dwarfism, infertility, epilepsy, and early mortality (12). This limits their use for conclusive studies about the cardiovascular roles of CNP. To overcome these limitations and unravel the possible role of endogenously formed local CNP in the regulation of cardiac fibrotic responses, we generated a mouse line that allows tamoxifen-inducible, Cre-mediated deletion of GC-B in fibroblasts. Our studies in these mice show that fibroblast-specific ablation of CNP/GC-B signaling results in excess cardiac fibrosis both in chronic Ang II exposure and after transverse aortic constriction (TAC). Intriguingly, these genotype-dependent changes were more prominent in female as compared with male mice.

## Results

### CNP stimulates GC-B/cGMP signaling in cultured human CFs and inhibits their activation.

**Figure 1:**
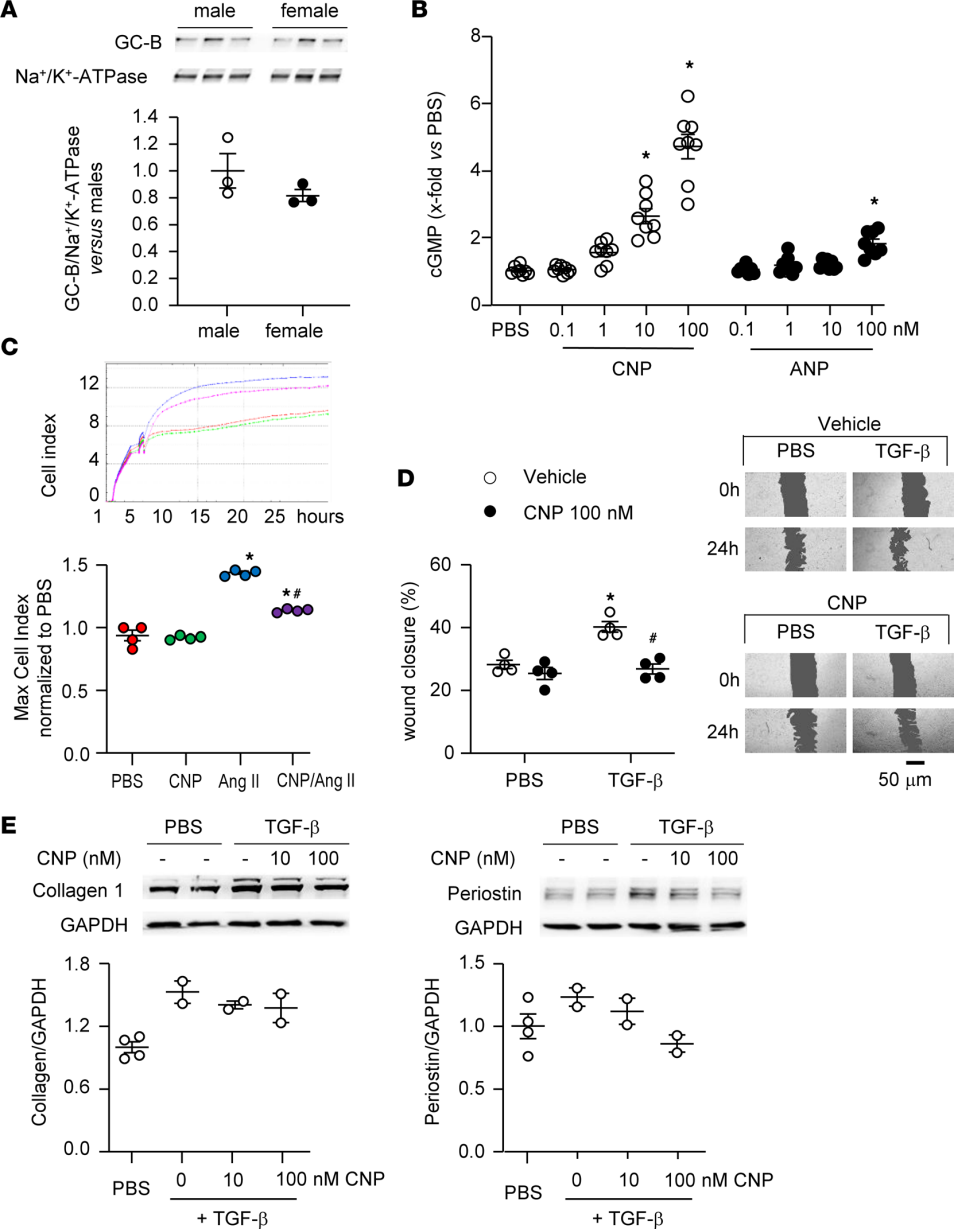
CNP/GC-B/GMP signaling in cultured human cardiac fibroblasts. Studies were performed with cells of 1 male and 1 female donor at passages 4 and 5. (**A**) Immunoblot: GC-B protein expression in enriched cell membranes was normalized to Na^+^/K^+^-ATPase and calculated as *x*-fold vs. male fibroblasts (*n* = 3 of each donor; unpaired 2-tailed *t* test). (**B**) Effects of CNP and ANP on intracellular cGMP contents determined by radioimmunoassay and calculated as *x*-fold vs. PBS; *n* = 8 (4 wells from each donor; 1-way ANOVA). (**C**) iCELLigence technology was used to study cell proliferation in real time. PBS, CNP (100 nM), and/or Ang II (10 nM) were added for 24 hours (*n* = 4 wells per condition, 2 from each donor; nonparametric Kruskal-Wallis analyses). (**D**) For wound closure assays, cells were seeded and grown until confluent. A scratch was made followed by TGF-β (10 ng/mL) treatment in the absence (vehicle) or presence of CNP (100 nM). Wound closure was quantified at 24 hours. Right: Representative images (*n* = 4; 2-way ANOVA). (**E**) Immunoblots: Expression of collagen I and periostin in cells treated with vehicle (PBS) or TGF-β (10 ng/mL) for 24 hours in the absence (PBS) or presence of CNP (10 and 100 nM). Target proteins were normalized to GAPDH (as loading control) and expressed as *x*-fold vs. PBS (*n* = 2–4 from the 2 donors; statistical evaluations were not applied due to low number of samples). *P < 0.05 vs. PBS; ^#^*P* < 0.05 vs. Ang II (**C**) or vehicle (**D**).

To examine the role of CNP in the regulation of cardiac fibrosis, we first studied GC-B/cGMP signaling in cultured human CFs. Cells from a male and a female donor were obtained through a commercial provider (see Methods) and studied at passages 4 and 5. Immunoblot studies demonstrated the expression of GC-B, with similar levels in CFs from both donors (Figure 1A). Accordingly, the baseline intracellular cGMP levels of confluent CFs were also similar: 80.7 ± 3.4 (male donor) and 72 ± 4.3 fmol/well (female). CNP increased cGMP levels in a concentration-dependent manner, whereas ANP barely had an effect (Figure 1B).

Recording cellular impedance with xCELLigence technology, we evaluated whether CNP modulates cell proliferation. As shown in Figure 1C, Ang II (100 nM, 24 hours) increased the proliferation rate of CFs by 1.5-fold. CNP (100 nM) did not affect baseline proliferation but significantly reduced the effect of Ang II. To assess CF migration in wound closure assays, cells were seeded in 24-well plates and grown until confluent. A scratch was made followed by TGF-β (10 ng/mL) treatment in the absence (vehicle) or presence of CNP (100 nM). Wound closure was quantified at 24 hours and showed that the stimulatory effect of TGF-β was fully prevented by CNP (Figure 1D and Supplemental Figure 1; supplemental material available online with this article; https://doi.org/10.1172/jci.insight.160416DS1). Moreover, treatment of CFs with TGF-β (10 ng/mL, 24 hours) increased the expression of the ECM proteins collagen 1 and periostin and this effect was also attenuated by CNP (immunoblots in Figure 1E). However, due to the small sample number this last experiment was not suitable for statistical evaluation.

### Infusion of a low CNP dose attenuates Ang II–induced cardiac remodeling.

**Figure 2:**
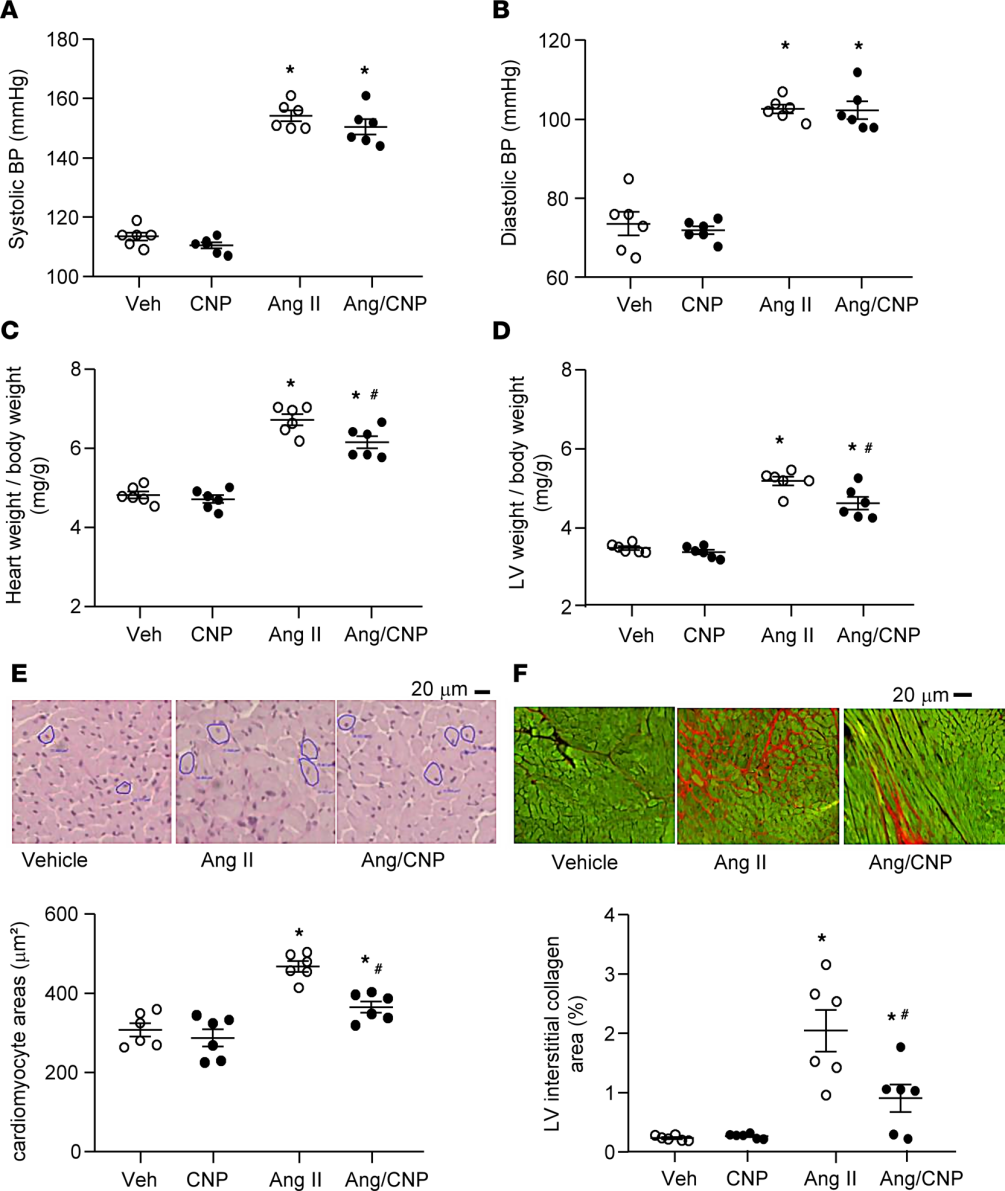
Cardiovascular effects of CNP and/or Ang II infused into wild-type mice with subcutaneous osmotic minipumps for 14 days. (**A**) Systolic blood pressure (BP). (**B**) Diastolic BP. (**C**) Heart weight–to-BW ratios. (**D**) Left ventricular (LV) weight–to-BW ratios. (**E**) LV cardiomyocyte cross-sectional areas determined on PAS-stained sections. (**F**) Interstitial collagen fractions determined on Sirius red–stained sections. *n* = 6 mice per group. *P < 0.05 vs. vehicle (Veh); ^#^*P* <0.05 vs. Ang II (2-way ANOVA followed by Tukey’s multiple-comparison test).

We next determined the effect of exogenous *s*ynthetic CNP on Ang II–induced cardiac fibrosis. CNP (0.05 μg/kg/min) and/or Ang II (1,000 ng/kg BW/min) were infused into wild-type C57BL/6N mice via osmotic minipumps over 2 weeks. We previously determined that this CNP dose mildly raises circulating plasma levels by approximately 1.5-fold (13). Tail cuff recordings of blood pressure in awake trained mice confirmed that Ang II provoked arterial hypertension (Figure 2, A and B). This was accompanied by LV hypertrophy (as judged by the heart and LV weight–to–body weight (BW) ratios and cardiomyocyte cross-sectional areas illustrated in Figure 2, C–E) and augmented LV interstitial collagen deposition (Figure 2F). Infusion of CNP did not change resting blood pressure or the hypertensive actions of Ang II (Figure 2, A and B). Despite this, the cardiac hypertrophic and fibrotic effects of Ang II were significantly attenuated by CNP (Figure 2, C–F).

### Development and characterization of a fibroblast-specific GC-B–KO mouse.

Our data so far demonstrate that exogenous CNP stimulates GC-B/cGMP signaling in CFs and attenuates the effects of Ang II and TGF-β on CF proliferation and migration (in vitro) as well as on cardiac remodeling (in vivo). To investigate whether endogenous paracrine CNP/GC-B signaling regulates the activity of CFs, we crossed mice with a floxed GC-B gene (*Npr2^fl/fl^*; ref. 14) with *Col1a2*-*Cre^ERT2^* mice carrying a tamoxifen-inducible Cre recombinase element under the fibroblast-specific regulatory sequence of the proα2 type 1 collagen gene (15). Such Cre mice have been used to induce Cre*-lox*–mediated deletion of genes specifically in fibroblasts (15), including those in the heart (16–19), without affecting the targeted gene in other types of cells (15–19). At the age of 4 weeks, *Npr2^fl/fl^* mice with or without the *Col1a2-Cre^ERT2^* transgene were injected with tamoxifen for 5 days and experiments were started 2 weeks later.

**Figure 3:**
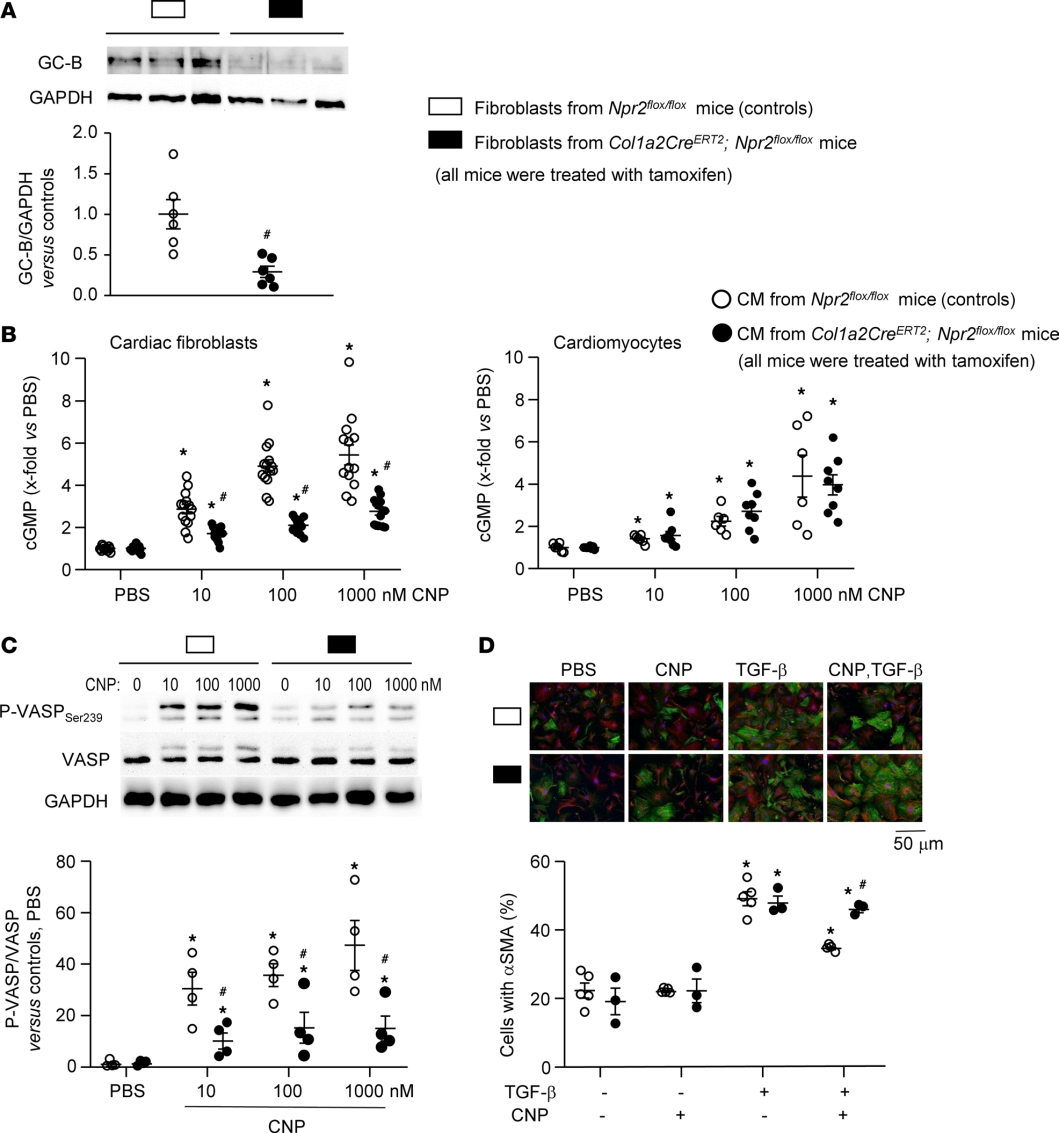
Comparison of GC-B expression and signaling in cardiac fibroblasts isolated from tamoxifen-treated *Npr2^fl/fl^* mice (controls) and *Npr2^fl/fl^;Col1a2-Cre^ERT2^* littermates. Cells were studied at passages 3 and 4. (**A**) Immunoblot: Expression of GC-B was normalized to GAPDH and calculated as *x*-fold vs. controls (*n* = 6 from 3 mice per genotype and sex; unpaired 2-tailed Student’s *t* test). (**B**) Effects of CNP on intracellular cGMP contents of cardiac fibroblasts (left; *n* = 14) and myocytes (right panel; *n* = 6–8) were determined by radioimmunoassay and calculated as *x*-fold vs. PBS. Baseline cGMP values: control fibroblasts, 121.9 ± 9.4 fmol/well; KO fibroblasts, 128.0 ± 3.2 fmol/well; cardiomyocytes from control mice: 14.8 ± 1.8 fmol/well; cardiomyocytes from KO mice: 17.0 ± 2.0 fmol/well. CM, cardiomyocytes. (**C**) Immunoblot: Effect of CNP on the phosphorylation of vasoactive stimulated phosphoprotein (p-VASP) at position Ser_239_ in fibroblasts. p-VASP was normalized to total VASP and calculated as *x*-fold vs. PBS (*n* = 4). (**D**) Immunocytochemistry: Effects of 100 nM CNP and/or 10 nM TGF-β (24-hour incubation) on the proportion of α-SMA–expressing cells (5 control and 3 KO samples per condition). *P < 0.05 vs. baseline (PBS); ^#^*P* < 0.05 vs. controls by 2-way ANOVA followed by Tukey’s multiple-comparison test (**B**–**D**).

To study the efficiency and selectivity of the deletion, CFs and cardiomyocytes were isolated from such tamoxifen-treated *Npr2^fl/fl^* and *Npr2^fl/fl^;Col1a2-Cre^ERT2^* littermates. Cultured CFs were passaged twice and then plated for experiments. Western blotting demonstrated an approximately 75% reduction in GC-B expression in CFs from *Npr2^fl/fl^;Col1a2-Cre^ERT2^* mice (Figure 3A). Despite that, the baseline cGMP levels were not different between CFs from *Npr2^fl/fl^* (122 ± 9.4 fmol/well) and *Npr2^fl/fl^;Col1a2-Cre^ERT2^* mice (128 ± 3.2 fmol/well). Also, the cGMP levels of freshly isolated cardiomyocytes did not differ between genotypes: 14.8 ± 1.8 fmol/well and 17 ± 2 fmol/well, respectively. CNP concentration-dependently increased cGMP contents of cultured CFs and freshly isolated cardiomyocytes from *Npr2^fl/fl^* mice (Figure 3B). As also shown, CNP/cGMP effects were preserved in cardiomyocytes but greatly diminished in CFs from *Npr2^fl/fl^;Col1a2-Cre^ERT2^* mice. Accordingly, in *Npr2^fl/fl^* fibroblasts CNP (10–1,000 nM, 15 minutes) stimulated the phosphorylation of the cytoskeleton-associated vasodilator-stimulated phosphoprotein (VASP) at Ser_239_, the specific target site of cGMP-dependent protein kinase I (cGKI). This effect was markedly and significantly reduced in CFs from *Npr2^fl/fl^;Col1a2-Cre^ERT2^* mice (Figure 3C). Incubation with TGF-β (10 ng/mL for 24 hours) enhanced the proportion of α-SMA–positive CFs (immunocytochemistry; Figure 3D). CNP (100 nM) significantly attenuated this effect of TGF-β in *Npr2^fl/fl^* but not in *Npr2^fl/fl^;Col1a2-Cre^ERT2^* fibroblasts (Figure 3D). Based on these results, we used the nomenclature “control” and “Fibro GC-B–KO” for tamoxifen-treated mice of these 2 genotypes, respectively.

**Figure 4:**
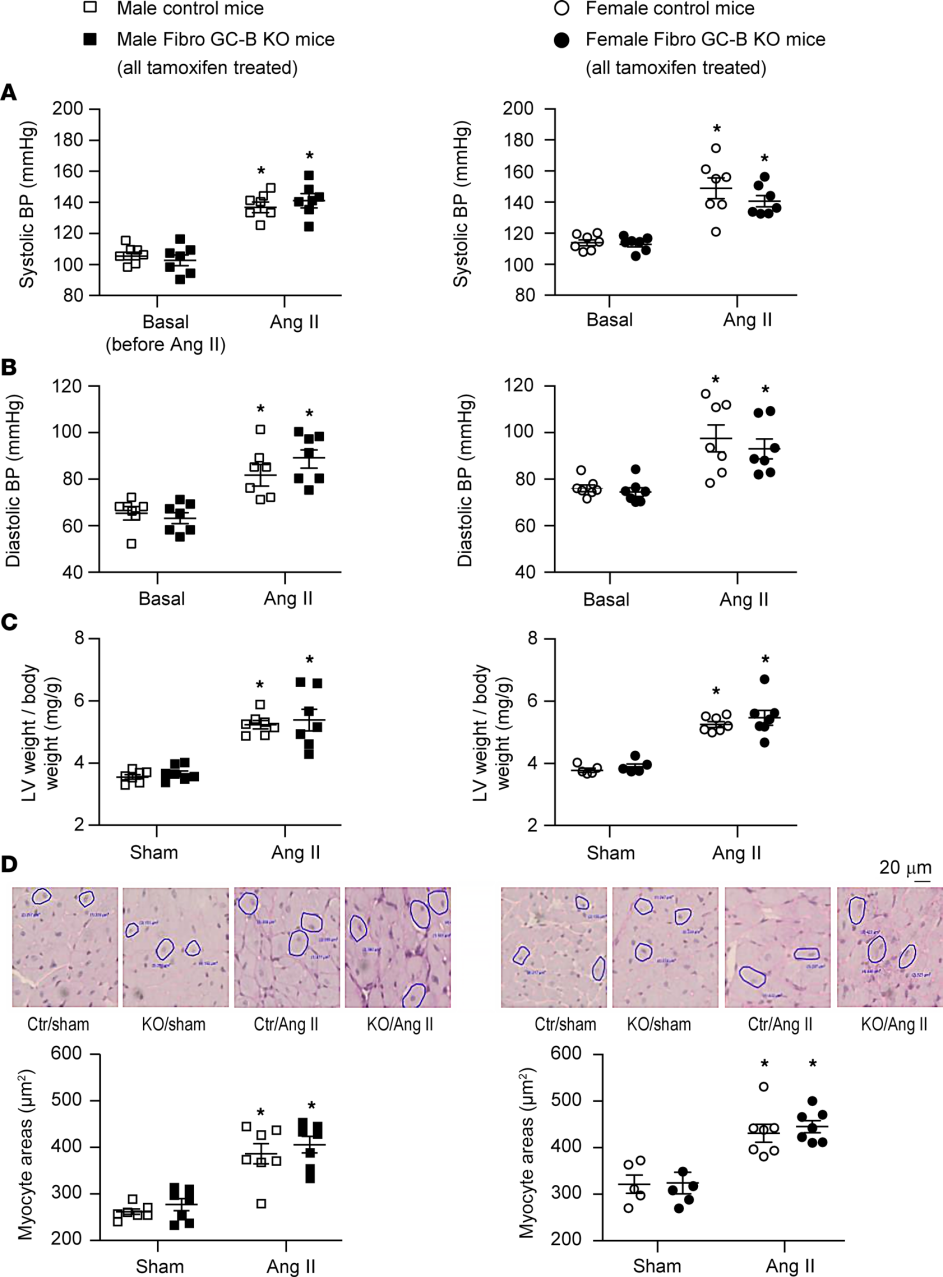
Effects of Ang II infusion via osmotic minipumps for 2 weeks on arterial blood pressure and cardiomyocyte sizes of control and Fibro GC-B–KO littermates. Left panels illustrate data of male mice; right panels illustrate data of females. (**A** and **B**) Systolic and diastolic blood pressure (BP) levels determined in trained awake mice via tail cuff; 7 mice of each sex and genotype were studied before (basal) and during the last 5 days of Ang II treatment. (**C**) Left ventricular (LV) weight–to-BW ratios. (**D**) LV cardiomyocyte cross-sectional areas were determined by morphometrical analyses of PAS-stained sections. Males: 7 sham controls, 7 sham KO, and 7 Ang II–treated mice per genotype. Females: 5 sham mice and 7 Ang II–treated mice per genotype (**C** and **D**). *P < 0.05 vs. sham; ^#^*P* < 0.05 vs. control mice (2-way ANOVA followed by Tukey’s multiple-comparison test).

**Figure 5:**
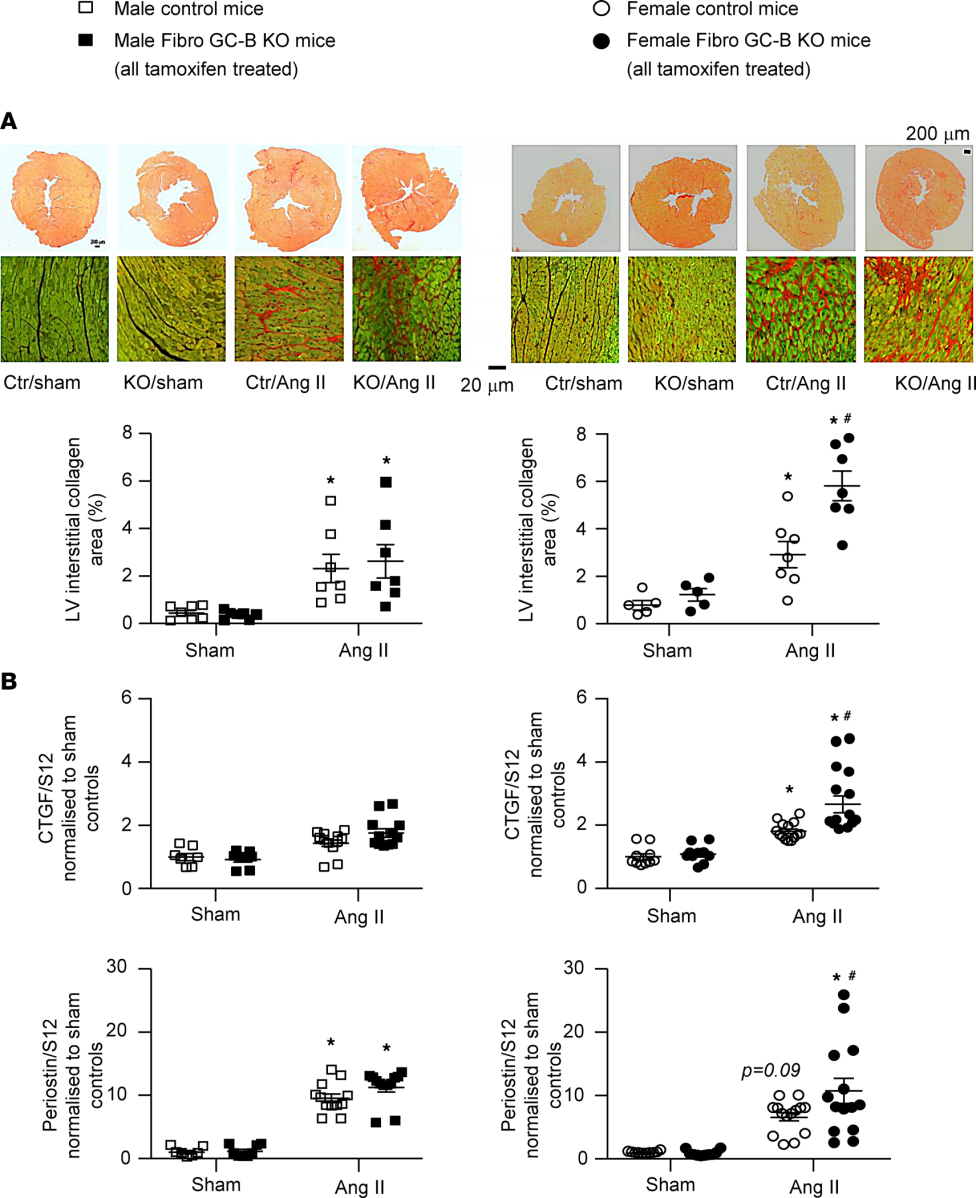
Cardiac fibrotic effects of Ang II (infused via osmotic minipumps for 2 weeks) in control and Fibro GC-B–KO male and female mice. Left panels: Data from males; right panels: data from females. (**A**) The collagen areas were determined as fraction (%) of the total area of the corresponding Sirius red–stained LV section. *n* = 7 sham and 7 Ang II–treated males and 5 sham and 7 Ang II–treated females per genotype. (**B**) qRT-PCR analyses of LV mRNA expression of connective tissue growth factor (CTGF) and periostin. Values are the ratios of mRNA levels relative to S12, expressed as *x*-fold vs. sham mice. Studies were performed with 2 LV samples from each mouse. *n* = 8 samples from sham control, 8 from sham KO, and 12 from Ang II–treated male mice per genotype. *n* = 10 samples from sham and 14 from Ang II–treated female mice per genotype. *P < 0.05 vs. sham; ^#^*P* < 0.05 vs. control mice (2-way ANOVA followed by Tukey’s multiple-comparison test).

In contrast to mice with global GC-B deletion (2, 12), such Fibro GC-B–KO mice have normal Mendelian inheritance, life span, and skeletal growth. Under baseline and sham conditions, arterial blood pressure, cardiac and LV weights, cardiomyocyte cross-sectional areas, and cardiac interstitial collagen fractions were not different between control and KO littermates from both sexes (please see Results for basal and sham conditions in Figures 4 and 5). LV function was recorded by invasive pressure-volume loop measurements in anesthetized mice (closed chest) and showed normal LV contraction and relaxation (the results for sham control and sham Fibro GC-B–KO male and female mice are depicted in Supplemental Table 1, A and B).

### Female mice with fibroblast-restricted GC-B inactivation show enhanced cardiac fibrosis in response to Ang II.

To explore the effects of fibroblast-specific GC-B deletion on pathological cardiac remodeling, we treated control and Fibro GC-B–KO male and female mice with Ang II (1,000 ng/kg BW/min) via subcutaneous osmotic minipumps for 2 weeks. The hypertensive and LV hypertrophic actions of Ang II were not different between genotypes and sexes (Figure 4, A–D). In the group of male mice, the profibrotic effects of Ang II as judged from the LV interstitial collagen fractions (in percentage of Picrosirius red–stained section areas) were also similar in control and KO littermates (Figure 5A). Concordantly, the LV mRNA expression levels of the profibrotic genes encoding connective tissue growth factor (*CTGF*) and periostin were also not different between males of the 2 genotypes (Figure 5B). Cardiac output, LV ejection fractions, and the maximal rates of LV contraction and relaxation of control and KO mice were not altered by Ang II treatment (Supplemental Table 1A). However, LV end-diastolic pressures, the relaxation time constant (Tau, in ms) and the effective arterial elastance (E_a_, in mmHg/μL) were mildly increased by Ang II, indicating compromised ventricular filling due to myocardial stiffening. Such Ang II–induced changes in diastolic function were similar in male control and KO mice (Supplemental Table 1A).

In contrast to the male KO mice, the female Fibro GC-B–KO mice treated with Ang II exhibited significantly increased LV interstitial fibrosis as compared with controls (Figure 5A), despite the similar hypertensive and cardiac hypertrophic reactions. This was associated with augmented LV mRNA expression of CTGF and periostin (Figure 5B). In line with these morphological and molecular changes, LV end-diastolic pressure, E_a_, and Tau were greater in Ang II–treated KO as compared with control females, indicating enhanced passive stiffness (Supplemental Table 1B).

### Fibroblast-specific GC-B–deleted mice show enhanced cardiac remodeling and dysfunction after mild TAC.

Ang II infusion promotes cardiac remodeling both through increasing arterial blood pressure and cardiac afterload, and through direct effects on cardiomyocytes and CFs (20). The observation that synthetic CNP inhibits the proliferative effects of Ang II in cultured CFs (Figure 1C) raised the question whether the endogenous hormone specifically interferes with the direct profibrotic effects of Ang II (20), or also counterregulates the complex profibrotic pathways involved in hypertensive heart disease. To answer this question, we next investigated how ablation of CNP/GC-B signaling in fibroblasts would affect cardiac remodeling and function in a mouse model of mild LV pressure overload induced by TAC.

**Figure 6:**
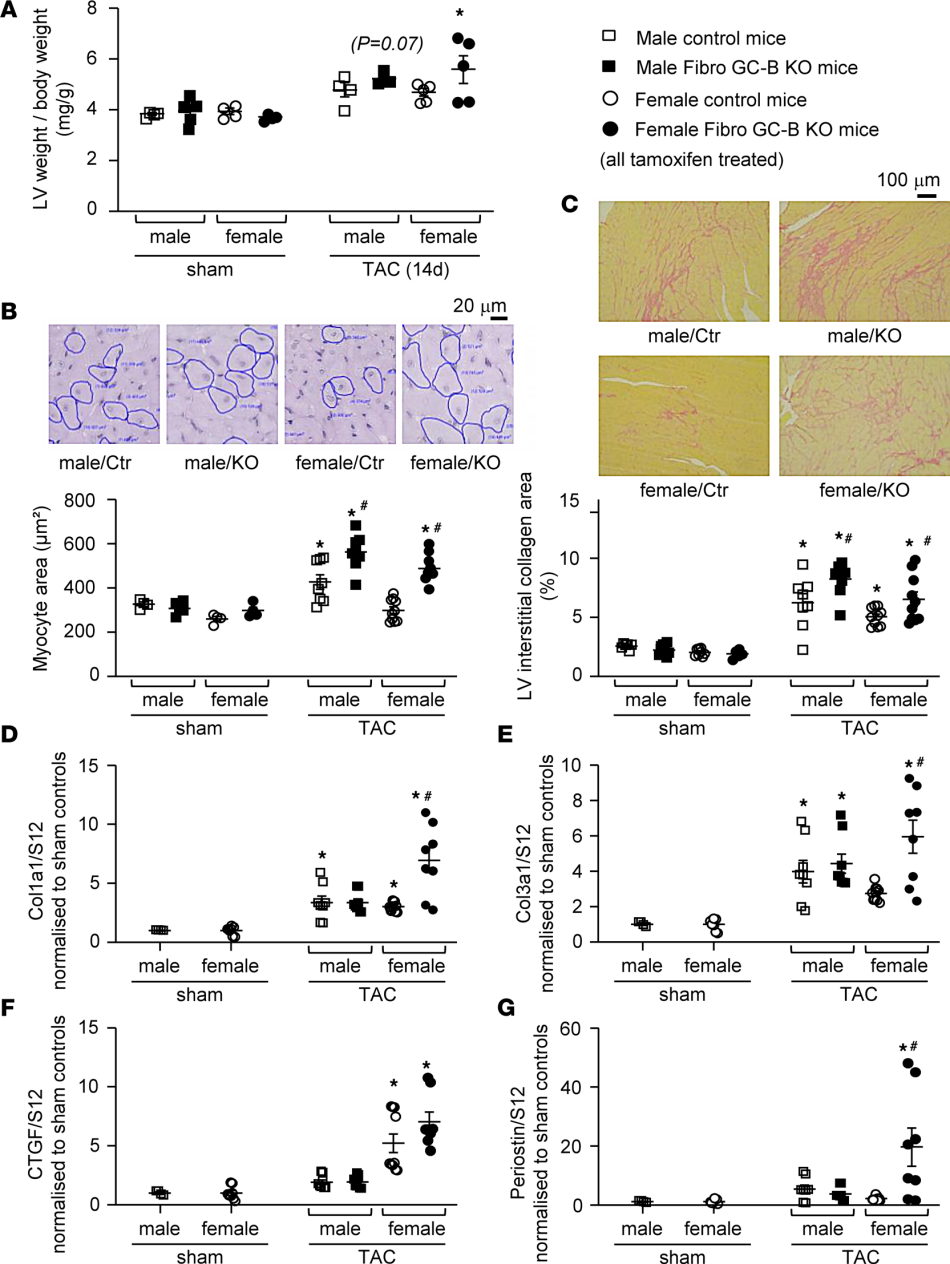
Effect of surgical transverse aortic constriction (TAC) for 14 days on cardiac remodeling of Fibro GC-B–KO and control littermate mice. (**A**) Left ventricular (LV) weight–to-BW ratios. Sham: 4 control and 5 KO males, 4 control and 4 KO females. TAC: 4 males and 5 females per genotype. (**B**) Cardiomyocyte cross-sectional areas were determined on PAS-stained LV sections. For sham, 1 section/heart and for TAC, 2 sections/heart were evaluated (~100 myocytes per section). (**C**) Interstitial collagen fractions were determined on 2 Sirius red–stained sections from each left ventricle. (**D**–**G**) qRT-PCR analyses of LV mRNA expression of collagen 1 (Col1a1), collagen 3 (Col3a1), connective tissue growth factor (CTGF), and periostin. Values are the ratios of mRNA levels relative to S12, expressed as *x*-fold vs. sham mice. Studies were performed with 2 LV samples from each mouse. Sham: 2 male and 4 female control mice; TAC: 4 control and 4 KO males, 5 control and 4 KO females. *P < 0.05 vs. sham, ^#^*P* < 0.05 vs. control mice (myocyte areas: 1-way ANOVA; all others: 2-way ANOVA followed by Tukey’s multiple-comparison test).

Invasive catheterizations in anesthetized male and female mice showed that after 14 days of TAC, the pressure load of the LV was increased by approximately 40 mmHg, without differences between genotypes and sexes (Supplemental Table 2, A and B). This resulted in a mild but significant increase in the LV weight–to-BW ratios, with a trend to greater values in the Fibro GC-B–KO mice (Figure 6A). Indeed, morphometric analyses of cardiomyocyte cross-sectional areas on periodic acid–Schiff–stained (PAS-stained) sections revealed that LV myocyte hypertrophy induced by TAC was significantly greater in male and female Fibro GC-B–KO mice as compared with their respective controls (Figure 6B). Moreover, morphometric analyses of Picrosirius red–stained LV histological sections showed that the profibrotic effects of TAC were also significantly increased in male and female KO mice in comparison with respective controls (Figure 6C). In line with cardiac interstitial fibrosis, the LV mRNA expression levels of the collagens I (*Col1a1*) and III (*Col3a1*) were increased in mice with TAC (as compared with sham) (Figure 6, D and E). As shown, whereas male control and KO mice showed similar responses, the female KO mice had significantly increased collagen I and III mRNA levels as compared with controls. CTGF mRNA levels barely increased in control and KO males with TAC, whereas they increased significantly in control and even more in KO females (Figure 6F). Moreover, only the female KO mice with TAC showed increased LV mRNA levels of periostin (Figure 6G).

**Figure 7:**
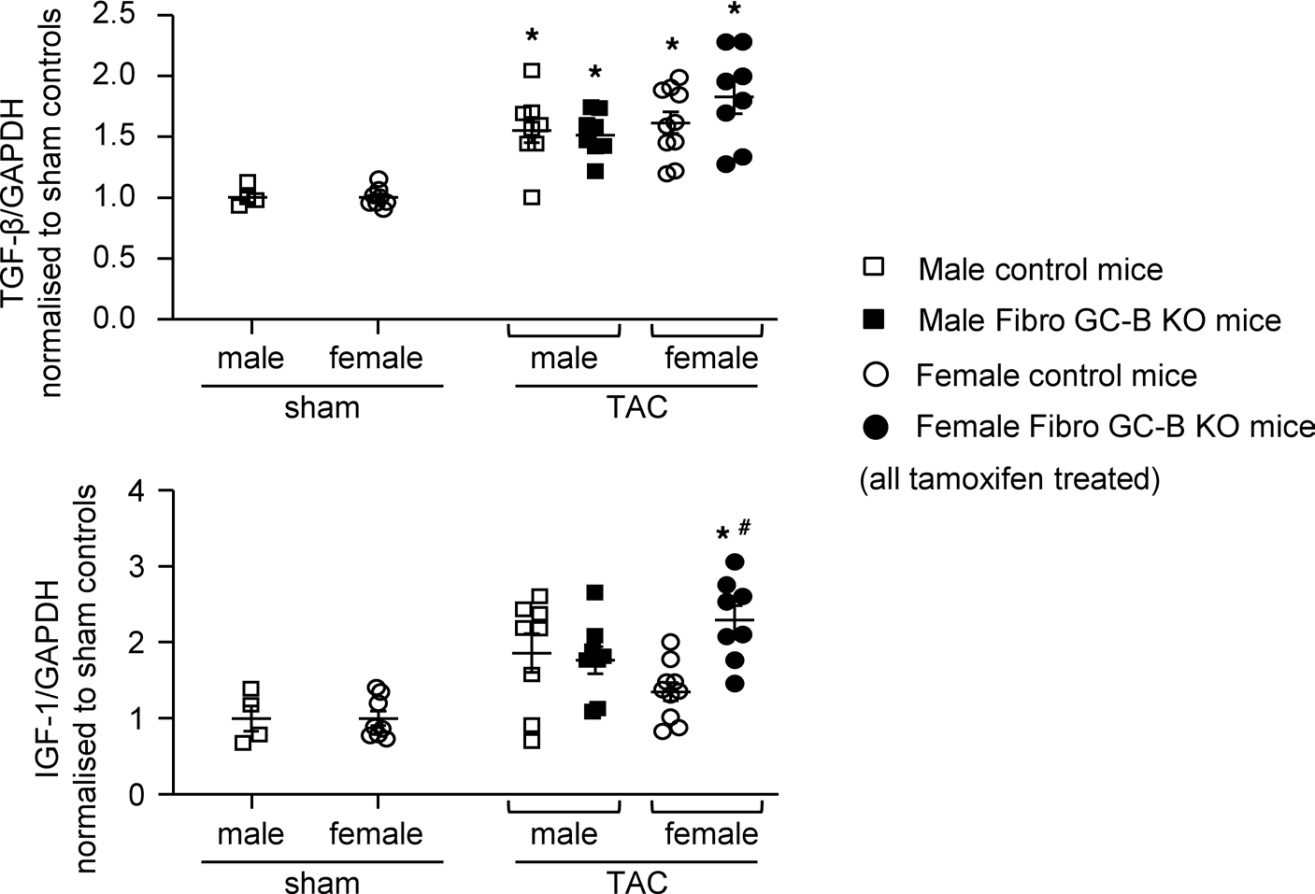
Effect of surgical transverse aortic constriction (TAC) for 14 days on cardiac expression of growth factors in Fibro GC-B–KO and control littermate mice. qRT-PCR analyses of LV mRNA expression of insulin-like growth factor-1 (IGF-1) and transforming growth factor-β (TGF-β). Values are the ratios of mRNA levels relative to GAPDH, expressed as *x*-fold vs. sham mice. Studies were performed with 2 LV samples from each mouse. Sham: 2 male and 4 female control mice; TAC: 4 control and 4 KO males, 5 control and 4 KO females. *P < 0.05 vs. sham; ^#^*P* < 0.05 vs. control mice (2-way ANOVA followed by Tukey’s multiple-comparison test).

To follow up the observation that mice with fibroblast-specific GC-B deletion have enhanced TAC-induced cardiomyocyte hypertrophy, we also studied the LV mRNA expression of known prohypertrophic factors released from fibroblasts such as TGF-β and insulin-like growth factor 1 (IGF-1). Whereas the levels of TGF-β were similarly increased by TAC in both genotypes and sexes, the expression of IGF-1 was only significantly induced in the female KO mice (Figure 7).

**Figure 8:**
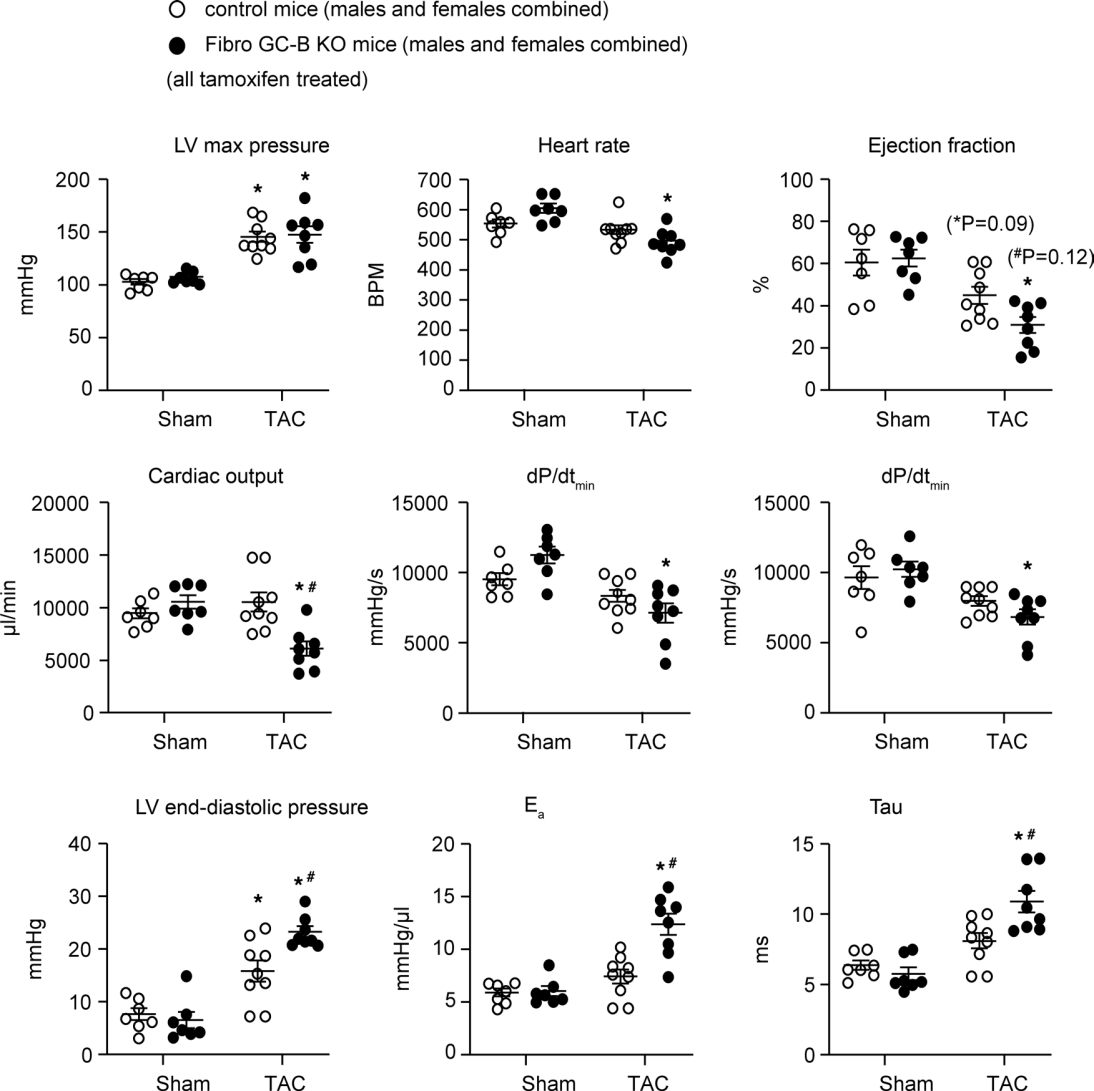
Left ventricular (LV) contraction and relaxation parameters of control and Fibro GC-B–KO mice were determined with pressure-volume catheters 2 weeks after sham or TAC surgeries. Female and male mice are shown together (Supplemental Table 2 provides these data separated by sex). Tau, LV relaxation constant [*T* = *P*/(d*P*/d*t_min_*)]; E_a_, effective arterial elastance, defined as the ratio of left ventricular end-systolic pressure (P_ed_) and stroke volume (SV). Sham: 7 mice per genotype; TAC: 9 control and 8 KO mice. *P < 0.05 vs. sham, ^#^*P* < 0.05 vs. control mice (LV end-diastolic pressure, E_a_ and Tau were evaluated by 1-way ANOVA; all others were evaluated with 2-way ANOVA followed by Tukey’s multiple-comparison test).

Supplemental Table 2 illustrates the impact of TAC on LV mechanical functions of male (top) and female mice (bottom). As shown, in control mice of both sexes such mild increases in afterload did not alter LV contraction and relaxation (controls with TAC in comparison with control sham mice). In contrast, the male and female KO mice with TAC had diminished cardiac output, ejection fractions, and maximal rates of LV contraction and relaxation. Moreover, LV end-diastolic pressures, E_a_, and Tau were significantly increased, indicating compromised ventricular filling due to severe myocardial stiffening. Since the cardiac parameters of TAC-operated control and KO mice did not differ between sexes, the combined results of males and females are illustrated in Figure 8, to highlight the genotype-dependent changes.

The results from our morphometric, molecular, and functional studies together demonstrate that Fibro GC-B–KO mice respond to TAC with increased cardiac hypertrophy, fibrosis, and failure. Such changes occurred in male and female KO mice, although they were more pronounced in the latter.

### CNP/GC-B signaling is greater in female as compared with male CFs.

**Figure 9:**
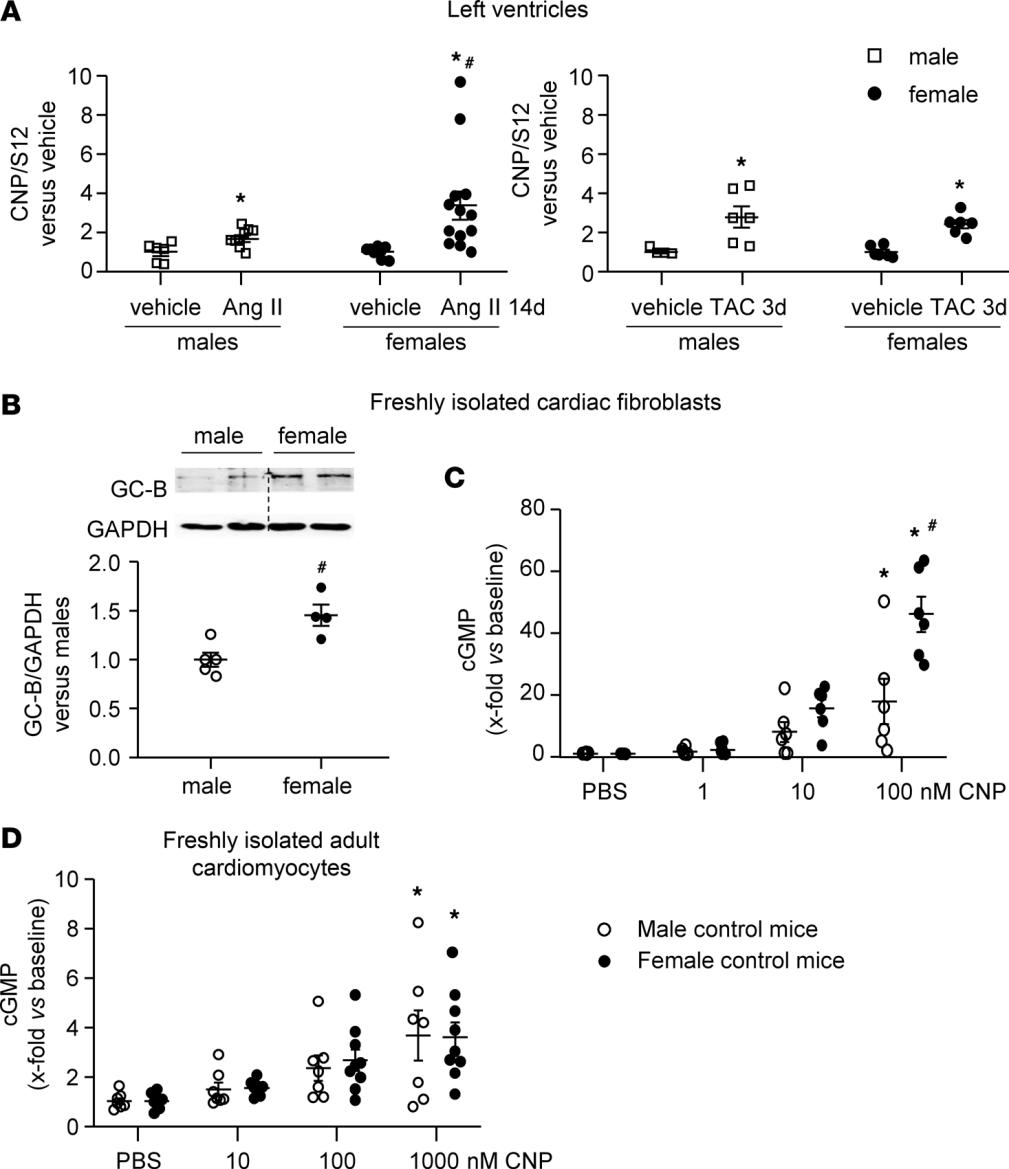
Comparison of CNP expression in left ventricles and of GC-B expression and cGMP signaling in freshly isolated cardiac fibroblasts and myocytes from male and female control mice (all P0). (**A**) Left ventricular (LV) CNP mRNA expression after Ang II infusion (left panel) or TAC (right panel). Values are the ratios of CNP mRNA level relative to S12, determined by qRT-PCR in 2 samples from each LV and expressed as *x*-fold versus samples from vehicle-treated mice of the same sex (Left panel: 6 samples from vehicle- and 10 samples from Ang II–treated males; 8 samples from vehicle and 13 samples from Ang II females. Right panel: 4 samples from sham and 6 from TAC males; 6 samples from 3 females per condition). (**B**) Immunoblot: GC-B protein expression in fibroblasts; levels were normalized to GAPDH and calculated as *x*-fold vs. males (*n* = 5 male and 4 female mice). (**C**) cGMP responses of such fibroblasts to CNP (6 samples from 3 mice per sex). Responses were calculated as *x*-fold increase in baseline values (basal: 16.5 ± 4.0 and 14.1 ± 0.3 fmol/well for male and female fibroblasts, respectively). (**D**) cGMP responses of cardiomyocytes to CNP (7 samples from 7 male mice; 9 samples from 9 female mice). Responses were calculated as *x*-fold increase in baseline values (basal: 0.18 ± 0.06 and 0.17 ± 0.07 fmol/well for male and female cardiomyocytes, respectively). *P < 0.05 vs. vehicle or PBS; ^#^*P* < 0.05 vs. male mice by 2-way ANOVA followed by Tukey’s multiple-comparison test (**A**, **C**, and **D**) or unpaired 2-tailed *t* test (**B**).

Our in vivo data demonstrated that inhibition of CNP/GC-B/cGMP signaling in CFs exacerbates the profibrotic actions of Ang II and TAC in male and especially in female mice. To characterize the mechanisms participating in this sex disparity, we compared CNP expression and signaling between male and female murine hearts. Because our previous studies had shown that the induction of CNP by TAC is acute and transient (13), here we studied cardiac CNP mRNA expression in mice with only 3 days of TAC. As shown in Figure 9A, Ang II infusions and TAC significantly raised LV CNP mRNA levels. The effects of Ang II were greater in females, whereas the effects of TAC were similar in both sexes.

As illustrated in Figures 1 and 3, we did not observe differences in GC-B expression and activity in cultured male and female CFs (human and murine). However, these studies were performed with passaged CFs, which may differ from CFs in situ. To generate “native” CFs, freshly isolated CFs were allowed to adhere to cell culture wells for 1 hour and were then directly lysed for immunoblots or used for cGMP determinations. Interestingly, GC-B expression levels were higher in female than in male CFs (Figure 9B), despite the fact that the basal cGMP values of such freshly plated CFs did not differ between sexes: 16.5 ± 4 fmol/well in male CFs, and 14.1 ± 0.3 fmol/well in female CFs. However, the cGMP responses to CNP were greater in female as compared with male CFs (Figure 9C). It is also noteworthy that such responses were overall greater in unpassaged CFs as compared with passaged CFs: 100 nM CNP raised the cGMP levels of the former by 17.94 ± 7.33-fold (male CFs) and 46.22 ± 5.34-fold (female CFs); in passaged cells the same CNP concentration increased the cGMP levels by only 4.63 ± 0.35-fold (human CFs, Figure 1B) and 4.91 ± 0.30-fold (murine CFs, Figure 3B). In comparison with unpassaged murine CFs, the cGMP responses of freshly isolated adult LV cardiomyocytes were much lower and not different in males and females (100 nM CNP increased cGMP contents by 2.35 ± 0.51-fold and 2.66 ± 0.43-fold in male and female cardiomyocytes, respectively) (Figure 9D).

## Discussion

Myocardial fibrosis provoked by ischemia, inflammatory reactions, or the neurohumoral imbalance accompanying arterial hypertension, results in cardiac stiffness and contractile dysfunction (1). Despite its prevalence, there is a lack of efficacious therapies. The renin–Ang II–aldosterone system is an important enhancer of cardiac fibrosis. Hence, the protective cardiovascular profile of drugs antagonizing the actions of aldosterone and Ang II is partly attributed to their antifibrotic effects (1). Herein, using complementary genetic and pharmacological approaches, we provide evidence that CNP, via GC-B, locally counteracts the activation of CFs by Ang II or chronic cardiac pressure overload. Consistent with this, inhibition (GC-B–KO) of CNP/GC-B/cGMP signaling in fibroblasts exacerbates the profibrotic actions of Ang II or TAC.

Intriguingly, in mice with ablated CNP/GC-B effects in CFs, a mild TAC not only provoked enhanced cardiac fibrosis but also enhanced cardiomyocyte hypertrophy. Moreover, cardiac systolic and diastolic functions were markedly impaired in such mice. In contrast, mice with a cell-specific deletion of GC-B in cardiomyocytes responded to TAC-induced pressure load with enhanced titin-based myocyte stiffness, but without enhanced hypertrophy or systolic dysfunction (13). Together, our previous (13) and present observations indicate that CNP modulates the intercellular fibroblast-to-cardiomyocyte communication and that CFs mediate both the antifibrotic and antihypertrophic effects of CNP. The existence of an intensive fibroblast-cardiomyocyte connection was already indicated by previously published studies. Concomitant attenuation of pathological cardiac fibrosis and myocyte hypertrophy was observed in mice with fibroblast-restricted deletion of profibrotic signaling molecules, such as TGF-β receptors 1 and 2, Hsp47, or G protein–coupled receptor kinase 5 (GRK5) (21–23). It was suggested that fibroblast-dependent stiffening of the ECM is required for the hypertrophic response of cardiomyocytes (22). Prohypertrophic paracrine factors secreted from myofibroblasts, such as TGF-β, CTGF, and IGF-1 might also be involved (24, 25). Indeed, the cardiac mRNA levels of the ECM proteins collagen and periostin as well as of the growth factor IGF-1 were significantly increased in female Fibro GC-B–KO mice exposed to TAC and may have contributed to their enhanced hypertrophic response. However, male Fibro GC-B–KO mice had similar hypertrophy without the enhanced expression of such genes, indicating the participation of other mechanisms. The good fibroblast-cardiomyocyte connection chaired by CNP might be even more “intimate”: cGMP formed in fibroblasts in response to CNP is able to enter the cardiomyocytes via gap junctions and this might provide the molecular basis for CNP-induced cGMP-dependent antihypertrophic effects (26). In accordance with this hypothesis, the cGMP-responses to CNP are greater in CFs than in cardiomyocytes (present study and ref. 26). Further studies are needed to determine the exact mechanism(s) by which CF-mediated effects of CNP moderate cardiomyocyte growth.

In line with experimental studies of the role of endothelial CNP in the control of blood pressure (11), the results from our histological and molecular studies indicate that the role of CNP/GC-B signaling in the moderation of CF activity is greater in female than in male mice. Enhanced Ang II–induced expression of CNP in female hearts as well as enhanced GC-B expression and signaling in native female CFs might contribute to this sex disparity. In fact, transcriptomic profiling studies of murine hearts at rest and after Ang II infusions revealed robust sexually dimorphic gene expression differences in fibroblasts, which are the major sex hormone receptor–expressing cells in the heart (27). In response to Ang II, female and male CFs regulated distinct genes that are associated with ECM remodeling and with prohypertrophic cell-cell communication (27). Our in vivo and in vitro studies suggest that the impact of the CNP/GC-B/cGMP pathway on these responses is greater in female CFs. Such sex differences in CNP/GC-B expression and/or signaling might also be relevant in humans. At the age of 20–50 years, CNP plasma levels are higher in women than in men (28), which is possibly related to the stimulatory effects of the female sex hormone estradiol on CNP expression in endothelia and other types of cells (29–31). Although our experimental studies provide insights into the cardiac impact of these sex differences in CNP signaling, further work is required to demonstrate the specific molecular mechanisms.

As mentioned in the Introduction, previous studies in mice with conventional, systemic GC-B deletion suggested that the cardiac antifibrotic actions of CNP are entirely mediated by NPR-C (3). However, such global GC-B–KO mice have a very severe systemic phenotype that limits cardiovascular studies (12). By generating mice with conditional, fibroblast-restricted GC-B deletion, we were able to overcome this limitation and dissect the crucial role of GC-B in the counteraction of CF activation. This notion is supported by the recent observation that the myokine Musclin (also named osteocrin), which prevents the binding of ANP and CNP to NPR-C, attenuated myocardial fibrosis and cardiac dysfunction in mouse hearts subjected to pressure overload (32) or to experimental myocardial infarction (33). Mechanistically, Musclin enhanced the abundance of the NPs, thereby inhibiting fibroblast activation through protein kinase G signaling (32). Taken together, our studies do not exclude a role for NPR-C, but they correct the published concept that GC-B does not participate in the cardioprotective actions of CNP.

### Clinical outlook.

Elevated myocardial diastolic stiffness as characteristically observed in heart failure, especially in heart failure with preserved ejection fraction, results from both increased myocardial collagen content and impaired titin-based cardiomyocyte distensibility (34). Our studies in genetic mouse models with either cardiomyocyte- (13) or fibroblast-restricted deletion of GC-B reveal that endogenous local CNP, elevating intracellular cGMP levels in cardiomyocytes and especially in fibroblasts, targets both components of diastolic stiffness; it enhances the phosphorylation of titin and thereby titin-based cardiomyocyte distensibility (13) and it counteracts pathological profibrotic and prohypertrophic CF activation (present study). These actions of locally formed CNP together protect the heart from pathological remodeling and contractile dysfunction. Drugs augmenting the CNP/cGMP signaling pathway, for instance by mimicking CNP actions with synthetic designer peptides (35), by inhibiting neprilysin-mediated degradation of endogenous CNP (36), or by blocking the phosphodiesterase-mediated degradation of cGMP (37), may have cardioprotective effects.

### Study limitations.

Because the effects of fibroblast GC-B inhibition were evaluated after 2 weeks of neurohumoral (Ang II) stimulation or pressure overload (TAC), for future clinical application, further studies are necessary to examine whether the effects of endogenous CNP on myocardial fibrosis persist for longer-term follow-up periods. Vice versa, future studies of the cardiac cell-type-specific gene changes in Fibro GC-B–KO mice at early stages of pathological CF activation (e.g., a few hours or days after TAC) might provide insights into the mechanisms of the CF-mediated antifibrotic and antihypertrophic effects of CNP. Additional limitations are the fact that with the tamoxifen-induced *Col1a2-Cre^ERT2^* transgene we achieved a marked and significant inhibition but not a full inactivation of the GC-B gene in fibroblasts, and that this inhibition presumably was not restricted to the CF population but involves fibroblasts from other organs.

## Methods

### Generation of mice with conditional, fibroblast-restricted deletion of GC-B.

Details of the generation of mice with a floxed GC-B gene (*Npr2^fl/fl^*) were previously published (14). Homozygous *Npr2^fl/fl^* mice were crossed with the *Col1a2-Cre^ERT2^* line (15–19). Four-week-old *Col1a2-Cre^ERT2+/0^;Npr2^fl/fl^* and control littermates lacking the *Cre* transgene (*Npr2^fl/fl^*) were treated for 5 days with daily intraperitoneal injections of tamoxifen (1 mg/day, diluted in peanut oil) (Sigma-Aldrich) (14). Mice homozygous for floxed *Npr2* and heterozygous for the *Cre* transgene were studied. *Npr2^fl/fl^* littermates without *Cre^tg^* served as controls within each experiment. The following studies were performed with 7- to 10-week-old male and female littermate mice of mixed (C57BL/6, 129Sv) background.

### Genotypings.

Genotypings were performed by PCR of tail tip DNA using primers GC-B-1 (GGACGACCCATCCTGTGATA) and GC-B-2 (GTTACAAACAAAAGCAAGATAAATACC), which amplify a 519-bp fragment for the GC-B (*Npr2*) wild-type allele, a 660-bp fragment for the floxed allele, and a 149-bp fragment for the KO allele (14). Presence of the *Col1a2-Cre^ERT2^* transgene was detected by PCR using primers Cre1 (5′-GCTGCCACGACCAAGTGACAGCAA) and Cre2 (5′-GTAGTTATTCGGATCATCAGCTACAC) (400-bp band) (15).

### Animal studies.

Wild-type, C57BL/6N mice (study 1) as well as control and Fibro GC-B–KO littermates (study 2) received vehicle, Ang II (1,000 ng/kg BW/min; Bachem), and/or CNP (0.05 μg/kg BW/min; Bachem) for 2 weeks via subcutaneously implanted micro-osmotic minipumps (Alzet Model 1002) (38). Arterial blood pressure was measured in awake mice by tail cuff (14, 38).

Surgical TAC, for 14 days, or sham operations were performed as previously described (13, 38). The depth of the isoflurane anesthesia was checked by ensuring that noxious pinch stimulation of the hind paw, the forepaw, and the ear did not evoke any motor reflexes. After thoracotomy, the aorta was ligated (7-0 Prolene) between the innominate and left carotid artery with an overlying 27-gauge needle to generate a reproducible, discrete stenosis. After ligation, the needle was withdrawn. Sham mice underwent the same procedure without aortic ligation. Buprenorphine (0.05–0.1 mg/kg BW) was used for preemptive and postoperative analgesia.

Terminal closed-chest hemodynamic studies of aortic pressure and LV function were performed with a retrogradely inserted SPR-839 Mikro-Tip pressure-volume catheter (Millar Instruments) (13, 38). After 30 minutes of stabilization, calibrated pressure-volume loops were continuously recorded for 30 minutes under 2% isoflurane anesthesia. Mice were sacrificed under deep anesthesia, the hearts were weighed, and left ventricles were dissected. LV slices were frozen in liquid nitrogen (for protein or mRNA extraction) or fixed in 4% buffered formaldehyde (for histology).

### Histology and morphometry of cardiac sections.

For histological analysis, formaldehyde-fixed left ventricles were embedded in paraffin, and 5-μm sections were stained with hematoxylin and eosin, PAS (to discriminate cardiomyocyte cell borders), or 0.1% Picrosirius red (for collagen) (13, 38). Photomicrographs of the PAS-stained LV sections were taken with a 20× objective and were evaluated using a computer-assisted image analysis system (Olympus). The mean cardiomyocyte areas were calculated by measuring 100 cells with a centrally located nucleus per specimen (13, 38).

Collagen quantification was performed as previously described (39). Picrosirius red–stained tissues emit a specific red collagen fluorescence, while viable cells like cardiomyocytes, leukocytes, and erythrocytes show a strong green autofluorescence. Image addition of both channels results in an image with complete overlap with the brightfield image and therefore the whole tissue content in the field of view. Images were acquired using a Zeiss Z1m Imager fluorescence microscope with standard FITC and rhodamine filter sets (FITC: Ex 490 nm, Em 520 nm; rhodamine: Ex 550 nm, Em 570 nm) and ZEM 2011 SP-1 software (Zeiss) using a fixed exposure time of 250 ms for each channel, with no additional image enhancements. Acquired red and green channels were exported from zvi to jpg files. Further image processing was performed using Adobe Photoshop 7.0. At first, green and red channels were reduced to grayscale. Then the 2 images were merged using “apply image” with mode “addition.” For measurement of the area without tissue holes and empty spaces “Color range” (“acquired colors,” “tolerance” set to 88) was used to mark the total tissue area automatically. Value of the marked area was extracted from the histogram. To determine the area of background corrected collagen in the image the viable tissue (green channel) was subtracted from the collagen image (red channel) using “apply image” with mode “subtraction,” which resulted in the background-corrected collagen area. The area was marked and extracted as explained above. The relationship between the total tissue area without empty spaces and background-corrected collagen was defined as the relative collagen content in the field of view. Images of at least 6 different locations were analyzed in each section to determine the relative collagen content (expressed as a percentage), with the investigator blinded to the genotypes (for details of the procedure and evaluation, please see ref. 39).

### Studies of cardiac gene expression.

Total RNA was isolated from LV myocardium using TRIzol reagent (Life Technologies). After reverse transcription (Transcriptor First Strand cDNA Synthesis Kit, Roche), real-time RT-PCR was performed using a LightCycler Instrument (Roche) with a real-time fluorogenic detection system for a kinetic approach (13, 14). The quantitative data were calculated from the kinetic curve of the PCR by interpolation with a standard curve generated using known amounts of the target DNA (13).

The sequences of the primers and probes (all from Roche) are listed in Supplemental Table 3.

### Experiments with cultured human and murine fibroblasts and cardiomyocytes.

Human ventricular CFs (C-12375, Promocell) from a male donor (lot 475Z017.1, age 22 years, White) and a female donor (lot 436Z024.3, age 33, White) were grown in Fibroblast Growth Medium 3 (C-23025, Promocell). Experiments were performed with cells at passages 4 and 5 starved in Fibroblast Basal Medium 3 (C-23230, Promocell) containing 0.5% FCS (C-37340, Promocell).

Cell proliferation was monitored in real time with the iCELLigence system (ACEA Biosciences). Briefly, 5,000 CFs were seeded per well on impedance plates (E-Plate L8, ACEA Biosciences) and allowed to adhere for 2 hours prior to testing. The cells were treated with vehicle or 100 nM CNP for 30 minutes and thereafter with vehicle or 10 nM Ang II. Proliferation was monitored for 24 hours. Data are presented as maximal cell index (a unitless parameter, automatically derived from recorded impedance values in iCELLigence software), normalized to vehicle-treated (PBS) cells (40).

For the migration assay, human CFs at passages 4–5 were grown in 24-well plates until confluent. After 24-hour serum starvation, plates were scratched, washed, and then treated for 30 minutes with CNP or vehicle and thereafter with human TGF-β (AF-100-21C, Peprotech) or vehicle for 24 hours. Images were captured directly after scratching and 24 hours after treatment with an inverted light microscope (4× objective, Olympus) and the wound closure (in percentage of the initial scratch area) was assessed.

To obtain murine CFs, donor mice were sacrificed under deep isoflurane anesthesia and the hearts were rapidly cannulated. Cardiac myocytes and CFs were isolated by Liberase (Roche Diagnostics GmbH)/trypsin digestion (protocol PP00000125 from The Alliance for Cellular Signaling) (13, 38). Myocytes were plated for 4 hours on laminin-coated dishes. CFs were cultured in fibroblast medium (Cardiac Fibroblast Medium-3, Lonza) for 5 days before passaging. Experiments were performed with murine CFs at passages 3 and 4. As specified in the Results, we also performed experiments with fresh CFs plated for 1 hour and used without passaging.

For cGMP determinations, the cells were incubated at 37°C with the phosphodiesterase inhibitor 3-isobutyl-1-methylxanthine (0.5 mM; Sigma-Aldrich) for 15 minutes and thereafter with synthetic CNP, ANP, (Bachem), or vehicle (saline) for an additional 10 minutes (13, 38).

### Immunoblotting.

Cultured CFs were lysed with RIPA buffer of the following composition: 25 mM Tris-HCl pH 7.6, 150 mM NaCl, 1% NP-40, 1% sodium deoxycholate, and 0.1% SDS (89900, Thermo Fisher Scientific). SDS-PAGE and immunoblotting were performed as described previously (13, 38). The sources and dilutions of all used primary antibodies are depicted in Supplemental Table 4. Protein bands were visualized with enhanced chemiluminescence and quantified by densitometry (14).

### Immunofluorescence.

Murine CFs were seeded on coverslips overnight. The cells were serum starved for 24 hours and subsequently treated with CNP (100 nM) or PBS for 2 hours and thereafter with murine TGF-β (10 ng/mL; R&D Systems) or vehicle for additional 24 hours. Cells were fixed with 4% paraformaldehyde, permeabilized with 0.2% Triton X-100, and then stained with anti-vimentin and anti–α-SMA antibodies (Supplemental Table 4). The secondary antibodies were Cy3-labeled anti-rabbit and Alexa Fluor 488–labeled anti-mouse antibodies (Supplemental Table 4). Mounting medium containing DAPI was used to visualize the cell nuclei (SRC-038448, Dianova). Fluorescence micrographs were captured using an epifluorescence microscope (Zeiss Z1m Imager equipped with an Axiocam MRm camera). Per well, 200 cells were counted with ImageJ software (v2.0.0, NIH), and the results were expressed as percentage of α-SMA–positive fibroblasts.

### Statistics.

Results were analyzed using Prism 9 software (GraphPad) and are presented as mean ± SEM. Data were tested for normality (Shapiro-Wilk test) and equal variance (*F* test). A *P* value of less than 0.05 was considered statistically significant. The individual statistical tests and sample sizes for each set of data are provided in the legends of figures and tables.

### Study approval.

All animal studies were approved by the Animal Care and Use Committee of University of Würzburg (Regierung von Unterfranken, approval numbers 55.2 2532-2-135 and -2-481) and conformed with the *Guide for the Care and Use of Laboratory Animals* published by the US NIH (National Academies Press, NIH Publication no. 85-23, revised 2011) and the guidelines from Directive 2010/63/EU of the European Parliament on the protection of animals used for scientific purposes.

## Author contributions

FW, EPS, KM, H Schrader, KV, TP, LK, MA, DM, MS, H Schmidt, BVS, KŠS, KS, and HAB conducted experiments and/or acquired, analyzed, and revised the data. CPD kindly provided the *Col1a2-Cre^ERT2^* mice. MK designed the research studies, analyzed and interpreted the data, and prepared the manuscript. All authors have approved the final version of the manuscript and agree to be accountable for all aspects of the work. FW and EPS share first authorship because they designed, performed, and/or mentored the most relevant parts of the in vivo studies. FW was mainly responsible for the generation of the genetic mouse model and the TAC experiments in these mice as well as the design of all qRT-PCRs. FW also conducted the experiments with human CFs and performed or supervised statistical evaluations. EPS performed the Ang II studies in the Fibro GC-B–KO mice as well as experiments with murine CFs.

## Supplementary Material

Supplemental data

Supporting data values

## Version 1 05/25/2023

In-Press Preview

## Version 2 07/10/2023

Electronic publication
