# Supplementary material for: Ablation of C-type natriuretic peptide/cGMP signaling in fibroblasts exacerbates adverse cardiac remodeling in mice
Source: JCI Insight. 2023 Jul 10;8(13):e160416. doi: 10.1172/jci.insight.160416 (PMC10371345; doi:10.1172/jci.insight.160416)
Supplement: Supplemental data [file jciinsight-8-160416-s080.pdf]

Supplemental figure 1

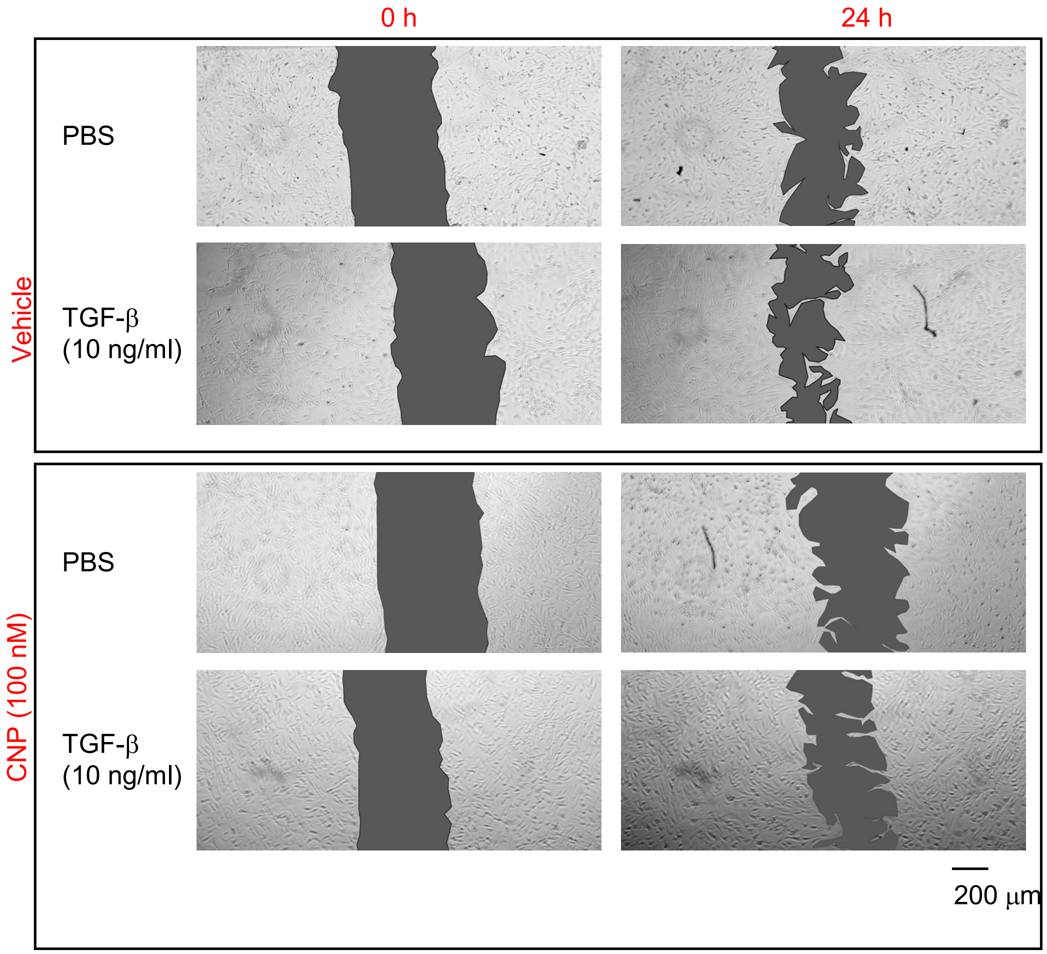

Supplemental Figure. Representative images of the wound closure assay in Human Cardiac Fibroblasts. CNP (100 nM) decreased TGF–β (10 ng/ml) induced wound closure (4 x Objective).

Supplemental Table S1a: AngiotensinII-treated male mice

Parameter	Controls Sham (4)	iFibro GC-B KO sham (4)	Controls Ang II (6)	iFibro GC-B KO Ang II (5)
Max aortic pressure (mmHg)	113 ± 2.4	111 ± 3.4	159 ± 1.77**	148 ± 6.3**
Heart rate (BPM)	617 ± 16	620 ± 8	648 ± 11	622 ± 21
Cardiac output (µl/min)	9596 ± 761	10320 ± 201	9319 ± 719	9852 ± 857
Ejection fraction (%)	64.2 ± 5.4	58.5 ± 2.9	58.8 ± 7.6	62.2 ± 4.7
LV max pressure (mmHg)	107 ± 3.3	108 ± 8	145 ± 3.7**	140 ± 6**
LV end-diastolic pressure (mmHg)	5.8 ± 0.06	5.7 ± 1.6	14.9 ± 3.1*	12.9 ± 2.7 (*P=0.07)
E_a (mmHg/µl)	6.62 ± 0.2	6.46 ± 0.09	10.1 ± 1*	7.9 ± 0.7*
dP/dt_{max} (mmHg/s)	11842 ± 601	12500 ± 463	13201 ± 525	12896 ± 746
dP/dt_{min} (mmHg/s)	-9523 ± 73	-10532 ± 546	-9555 ± 1073	-10061 ± 1249
Tau (ms)	5.24 ± 0.43	5.15 ± 0.5	7.36 ± 0.7*	7.02 ± 0.8 (*P=0.08)

Supplemental Table S1b: AngiotensinII-treated female mice

Parameter	Controls Sham (4)	iFibro GC-B KO sham (5)	Controls Ang II (6)	iFibro GC-B KO Ang II (6)
Max aortic pressure (mmHg)	109 ± 5	104 ± 4	144 ± 4.7**	145 ± 6.1**
Heart rate (BPM)	513 ± 49	539 ± 31	641 ± 12 (P=0.087)	612 ± 19 (*P=0.085)
Cardiac output (µl/min)	8604 ± 1033	7641 ± 634	9570 ± 551	7613 ± 961
Ejection fraction (%)	54.9 ± 2.7	49.5 ± 2.1	62 ± 2.6 (*P=0.09)	49 ± 5.3 ($^{\#}$P=0.07)
LV max pressure (mmHg)	103 ± 4.3	100 ± 3.4	138 ± 4.9**	134 ± 2.7**
LV end-diastolic pressure (mmHg)	5.9 ± 0.39	5.89 ± 0.6	13.8 ± 2.3*	21.5 ± 2.2**$^{\#}$
E_a (mmHg/µl)	6 ± 0.22	7.28 ± 1.09	9.1 ± 0.7**	13.8 ± 3.54
dP/dt_{max} (mmHg/s)	10036 ± 1526	8473 ± 544	14423 ± 791*	12861 ± 616**
dP/dt_{min} (mmHg/s)	-9160 ± 797	-8191 ± 857	-9233 ± 492	-8276 ± 560
Tau (ms)	6.55 ± 0.43	7.8 ± 0.9	7.02 ± 0.4	9.54 ± 0.8$^{\#}$

Supplemental Table S1. Hemodynamic indices derived from invasive left ventricular Millar catheter measurements in Ang II-treated control mice and littermates with fibroblast-specific deletion of the GC-B receptor (all tamoxifen treated). Tau: LV ventricular relaxation constant [$T = P/(dP/dt_{min})$]; E_a: effective arterial elastance, defined as the ratio of left ventricular end-systolic pressure (P_{ed}) and stroke volume (SV). *P<0.05 and **P<0.01 vs sham mice of same genotype; $^{\#}$P<0.05 vs AngII-treated control mice (heart rate, LV max pressure of male mice and heart rate, Ea and dP/dt_{min} of female mice were tested by One-Way-ANOVA; all others: Two-Way-ANOVA).

Supplemental Table S2a: male mice with TAC

Parameter	Controls Sham (3)	iFibro GC-B KO sham (4)	Controls TAC 14 d (4)	iFibro GC-B KO TAC 14 d (4)
Max aortic pressure (mmHg)	112 ± 4.0	112 ± 2.7	149 ± 6.1**	151 ± 6.1**
Heart rate (BPM)	547 ± 25	608 ± 19	547 ± 32	518 ± 21 (*P=0.09)
Cardiac output (µl/min)	9994 ± 769	10606 ± 1047	12202 ± 1743	6870 ± 1261#
Ejection fraction (%)	70.0 ± 6.9	59.1 ± 5.6	50.9 ± 6.2	30.0 ± 7.3*
LV max pressure (mmHg)	109 ± 1.4	110 ± 3.6	143 ± 3.9*	143 ± 9.6**
LV end-diastolic pressure (mmHg)	5.8 ± 1.6	5.8 ± 0.8	14.5 ± 4.2	22.9 ± 1.2**
E_a (mmHg/µl)	5.8 ± 0.7	6.3 ± 0.8	6.5 ± 1.2	11.5 ± 1.8*#
dP/dt_{max} (mmHg/s)	10582 ± 534	11188 ± 371	8449 ± 961	7061 ± 797**
dP/dt_{min} (mmHg/s)	-11627 ± 265	-10389 ± 339	-8106 ± 600*	-6978 ± 970*
Tau (ms)	5.7 ± 0.3	5.6 ± 0.6	7.5 ± 1.1	10.6 ± 1.2*

Supplemental Table S2b: female mice with TAC

Parameter	Controls Sham (4)	iFibro GC-B KO sham (3)	Controls TAC 14 d (5)	iFibro GC-B KO TAC 14 d (4)
Max aortic pressure (mmHg)	106 ± 2	108 ± 1	154 ± 8.0**	155 ± 5.8**
Heart rate (BPM)	564 ± 17	605 ± 31	526 ± 9	478 ± 19**
Cardiac output (µl/min)	9227 ± 667	10685 ± 818	9377 ± 452	5522 ± 559**##
Ejection fraction (%)	53.9 ± 8.3	68.0 ± 5.4	40.7 ± 5.1	32.1 ± 3.6**
LV max pressure (mmHg)	99 ± 3.3	106 ± 1.3	149 ± 8.5**	153 ± 13.3*
LV end-diastolic pressure (mmHg)	8.9 ± 1.4	7.5 ± 3.8	16.9 ± 1.8	23.7 ± 1.9**
E_a (mmHg/µl)	6.0 ± 0.5	5.6 ± 0.2	8.2 ± 0.7	13.2 ± 1.1**##
dP/dt_{max} (mmHg/s)	8823 ± 255	11452 ± 1452	8328 ± 355	7282 ± 1261*
dP/dt_{min} (mmHg/s)	-8273 ± 843	-10238 ± 1360	-7997 ± 418	-6843 ± 703 (*P=0.06)
Tau (ms)	6.9 ± 0.4	5.9 ± 0.8	8.6 ± 0.5	11.2 ± 1.1* (#P=0.08)

Supplemental Table S2. Hemodynamic indices derived from left ventricular Millar catheter measurements in TAC-treated male (Table S2a) and female (Table S2b) control mice and littermates with fibroblast-specific deletion of the GC-B receptor (all tamoxifen treated). Tau: LV ventricular relaxation constant [$T = P/(dP/dt_{min})$]; E_a: effective arterial elastance, defined as the ratio of left ventricular end-systolic pressure (P_{ed}) and stroke volume (SV). (n, numbers in the table). *P<0.05 and **P<0.01 vs sham mice of same genotype; #P<0.05 and ##P<0.01 vs TAC-treated control mice (max aortic pressure, LV max pressure, Ejection fraction and dP/dt_{min} of the male mice and LV end-diastolic pressure as well as Ejection fraction of female mice were tested by One-Way-ANOVA; all other parameters: Two-Way-ANOVA).

Supplementary Table S3. Primer sequences and Probes used for qRT-PCR.

**Target**	**Primer sequences (5'-3')**		**Universal probe library (Roche)**
**CNP**	Sense primer:<br>Antisense primer:	agcggtctgggatgttagtg<br>cgttggaggtgtttccagat	Probe 75<br>(catalog 046 889 880 01)
**Col1a1**	Sense primer:<br>Antisense primer:	catgttcagctttgtggacct<br>gcagctgacttcagggatgt	Probe 15<br>(catalog 046 851 480 01)
**Col3a1**	Sense primer:<br>Antisense primer:	catcaaaggacatcgaggattc<br>gtcctggactaccaattgcac	Probe 85<br>(catalog 046 890 970 01)
**CTGF**	Sense primer:<br>Antisense primer:	ccacccgagttaccaatgac<br>taggtgtccggatgcacttt	Probe 85<br>(catalog 046 890 970 01)
**FGF-2**	Sense primer:<br>Antisense primer:	cgacccacacgtcaaactac<br>cagccgtcctcttcctt	FastStart Essential DNA Green Master (Roche)
**GAPDH**	Sense primer:<br>Antisense primer:	atggtgaaggtcggtgtga<br>aatctccactttgccactgc	FastStart Essential DNA Green Master (Roche)
**IGF-1**	Sense primer:<br>Antisense primer:	cctgctgtgtaaacgacccgg<br>ggctgcttttgtaggcttcagtgg	FastStart Essential DNA Green Master (Roche)
**Periostin**	Sense primer:<br>Antisense primer:	cgggaagaacgaatcattaca<br>accttggagacctctttttgc	Probe 10<br>(catalog 04685091001)
**12S ribosomal RNA**	Sense primer:<br>Antisense primer:	gaagctgccaaggccttaga<br>aactgcaaccaaccaccttc	FastStart Essential DNA Green Master (Roche)
**TGF-β**	Sense primer:<br>Antisense primer:	tggagcaacatgtggaactc<br>cagcagccggttaccaag	Probe 72<br>(catalog 046 889 530 01)

Supplementary Table 4. Antibodies.

Target antigen	Vendor or Source	Catalog #	Working dilution
**alpha-Smooth muscle actin (SMA)**	Abcam, Cambridge, United Kingdom	ab7817	1:500
**Collagen 1**	ProteinTech, Planegg-Martinsried, Germany	14695-1-AP	1:1000
**GAPDH**	Cell Signaling, Leiden, Netherlands	#2118	1:5.000
**GC-B**	provided by our coauthor Dr. Hannes Schmidt. Firstly described in: Ter-Avetisyan G, Rathjen FG, Schmidt H. Bifurcation of axons from cranial sensory neurons is disabled in the absence of Npr2-induced cGMP signaling. J Neurosci. 2014 Jan 15;34(3):737-47.	-	1:10.000
**Na^+/K^+-ATPase**	Abcam, Cambridge, United Kingdom	ab76020	1:10.000
**Periostin**	Novus Biologicals, Wiesbaden Nordenstadt, Germany	NBP1-30042	1:1000
**phosphorylated VASP (Ser239)**	Cell Signaling, Leiden, Netherlands	#3114	1:1000
**VASP**	Cell Signaling, Leiden, Netherlands	#3112	1:1000
**Vimentin**	Abcam, Cambridge, United Kingdom	ab92547	1:400
**Alexa 488-labelled anti-mouse**	Life Technologies, Darmstadt, Germany	A-11001	1:500
**Cy3-labelled anti-rabbit IgG**	Dianova, Hamburg, Germany	111-165-003	1:200
**Peroxidase-conjugated Goat anti-guinea pig IgG**	Dianova, Hamburg, Germany	106-035-003	1:5000
**Peroxidase-conjugated Goat anti-mouse IgG**	Dianova, Hamburg, Germany	115-035-1062	1:10000
**Peroxidase-conjugated Goat anti-rabbit IgG**	Dianova, Hamburg, Germany	111-035-144	1:10000
